# Second Meeting of the Champaign County Medical Society

**Published:** 1859-02

**Authors:** S. L. Bearce, C. H. Mills

**Affiliations:** President; Secretary


PROCEEDINGS OF THE SECOND MEETING OF THE CHAMPAIGN COUNTY MEDICAL SOCIETY.

At the adjourned meeting of the physicians of Champaign county, Dr. Somers being absent, Dr. C. A. Thompson was called to the chair.

The minutes of the preceding meeting being read, Dr. Swain, Chairman of the Committee on Constitution and By-Laws, submitted a report, which report was accepted, and the committee was discharged. The Constitution and By-Laws were adopted as reported, with some amendments.

The following resolution was offered by Dr. Mills:

*Resolved*, That the physicians now present are, and do hereby constitute members of the Champaign County Medical Society, upon signing the constitution and paying the fee of membership; which resolution was adopted.

Whereupon the following enrolled and became members of the Society:

| | | |
|---|---|---|
| S. W. Kincaid, | Wm. M. Goodwin, | R. H. Brown, |
| John Swain, | S. L. Bearce, | J. T. Miller, |
| S. K. Page, | C. A. Thompson, | C. H. Mills. |
| A. J. Crain, | H. C. Howard, | |

The committee on permanent officers for the Society, reported as follows:

| | |
|---|---|
| *President*, . . . . . . . | Dr. S. L. Bearce. |
| *Sen. Vice-President*, . . | " R. H. Brown. |
| *Jun. Vice-President*, . . | " J. T. Miller. |
| *Recording Secretary*, . . | " C. H. Mills. |
| *Cor. Secretary*, . . . . . | " A. J. Crain. |
| *Treasurer*, . . . . : . | " S. K. Page. |

Who were elected, upon ballot, to fill the several offices, as reported by committee on officers.

On motion, Dr. Mills and Dr. Crain were appointed to wait upon the President elect to the chair.

The President appointed the following persons a Committee of Arrangements, and *ex officio* Censors: Drs. Mills, Kincaid, C. A. Thompson, Crain, and Swain.

Also the following as a Committee on Finance: Drs. Howard, Miller, and Goodwin.

The President appointed a Committee on Ethics, as follows: Drs. Kincaid, Brown, and Mills.

On motion, the President appointed a committee of three, to report a fee bill, at the next meeting of the Society, as follows: Drs. Swain, Miller, and Crain.

On motion, the President appointed Dr. C. A. Thompson a special committee of one, to write an Essay on Pneumonia, to be read at our next meeting.

On motion, Dr. Mills was selected by the Chair to write an essay upon some subject connected with surgery, and read it at the next meeting of the Society.

Dr. Crain was appointed a special committee to write upon some subject on Theory and Practice, and read at the next meeting.

On motion of Dr. C. A. Thompson, it was *Resolved*, That when we adjourn, we adjourn to meet at Urbana, on the first Friday of March.

Dr. Crain offered the following resolution, which was carried:

*Resolved*, That the thanks of the Society are hereby tendered to Messrs. Dr. Mills, McKinley & Jones, for the use of their office.

Dr. C. A. Thompson handed in the application of Dr. Somers, for membership. Also, Dr. Crain that of Dr. Hawes.

On motion of Dr. Miller, it was *Resolved*, That the proceedings of this meeting be handed to the *Urbana Union*, *Our Constitution*, and the *Chicago Medical Journal*, for publication.

On motion of Dr. Kincaid, the meeting adjourned.

S. L. BEARCE, M.D., *President*.

C. H. MILLS, M.D., *Secretary*.

